# Tuberculosis Transmission in a Primary School and a Private Language School. An Estimation of Infectivity

**DOI:** 10.3389/fped.2020.00010

**Published:** 2020-02-07

**Authors:** Sara Debulpaep, Alexandra Dreesman, Violette Dirix, Veronique Toppet, Maryse Wanlin, Lies Geysens, Wouter Arrazola de Oñate, Maryse Fauville, Françoise Mascart, Jack Levy, Françoise Mouchet

**Affiliations:** ^1^Pediatric Department, CHU Saint Pierre University Hospital, Université Libre de Bruxelles, Brussels, Belgium; ^2^Laboratory of Vaccinology and Mucosal Immunity, Université Libre de Bruxelles, Brussels, Belgium; ^3^Pediatric Department, Ghent University Hospital, Ghent, Belgium; ^4^Department of Pediatric Radiology, CHU Saint Pierre University Hospital, Université Libre de Bruxelles, Brussels, Belgium; ^5^French Association for Respiratory Health and Tuberculosis Control FARES, Brussels, Belgium; ^6^Flemish Association for Respiratory Health and Tuberculosis Control VRGT, Brussels, Belgium; ^7^The Belgian Scientific Institute for Public Health (Sciensano), Brussels, Belgium; ^8^Immunobiology Clinic, Hôpital Erasme, Université Libre de Bruxelles, Brussels, Belgium

**Keywords:** tuberculosis, children, contact screening, school, infectivity, transmission

## Abstract

**Introduction:** Belgium is a country with low incidence of tuberculosis (TB) and a very low number of TB cases in children. Children in contact with an adult smear-positive TB case are at high risk of transmission. Early diagnosis is important as young children have a significant predisposition of developing TB disease. In this paper, we describe two outbreaks after exposure to, respectively, two teachers with smear-positive pulmonary TB: one in a primary school, a nursery teacher, and another in a private language school.

**Methods:** An exposure investigation was carried out in both index cases household and school, according to the stone-in-the-pond principle. The tuberculin skin test (TST) was used a screening tool. The time elapsed between TB diagnosis in the index case and contact investigation was, respectively, 1 and 3 weeks. If this initial test was negative, it was repeated after a “window period” of ≥8 weeks.

**Results:** Index cases showed a transmission rate of, respectively, 13 and 40% in their classes at school, defined as casual contacts. The proximity of contact increased the risk of infection. TB disease was observed in, respectively, 4 and 11% of all the casual contacts; all of them were children younger than 5 years old. TB-infected and children with active TB disease had good compliance with recommended treatment. Uptake of chemoprophylaxis during the “window period” was poor, respectively, only 32–42%, in children under 5 years with an initially negative TST.

**Discussion:** The World Health Organization recommends to screen all young children (<5 years old) who have close contact with a person affected by pulmonary TB and to initiate Latent tuberculosis infection treatment even before infection can be demonstrated, after ruling out active TB disease. Despite this knowledge, a small percentage of the children younger than 5 years with no proof of infection was treated with the proposed chemoprophylactic treatment, in both cases.

**Conclusion:** This exposure investigation of two teachers detects high transmission among family contacts and school casual contacts. Recommendations for chemoprophylactic treatment in children <5 years showed low compliance, reflecting the difficulty of communication to staff, parents, and children in a school outbreak. It is essential to develop a new approach for this vulnerable group of patients. This approach could be improved, applied, and evaluated by National TB Control Programs, involving public and private health services. Public health authorities play a role in raising public awareness about the risks of TB for young children.

## Introduction

Belgium is a country with a low incidence of tuberculosis (TB) and a very low number of cases in children. In 2016, TB disease was reported in 27 children younger than 5 years old. Active TB in the pediatric population is most frequently detected by contact screening of adult TB cases (52% compared to 5.3% overall in 2016) (1). The source of infection among children is most often household members, even though transmission outside the home can occur (2, 3). Early diagnosis is essential because children aged <5 years old are at high risk of developing a clinical illness, those <2 years old risk developing even more severe diseases (4–6). Treatment of latent tuberculous infection (LTBI) reduces the risk of disease progression, especially in young children (3, 7). Children <5 years old, with exposure to sputum smear-positive tuberculosis, should be proposed chemoprophylactic treatment once TB disease has been ruled out. Chemoprophylactic treatment is a preventive therapy prescribed without definitive proof that latent infection is acquired, even if the result of a tuberculin skin test (TST) and/or an interferon gamma release assay (IGRA) is negative. This treatment can be stopped if the repeat TST remains negative after a “window period” (8, 9).

In this paper, we study the recent transmission of TB involving two schools in Belgium. We describe the contact screening and the clinical presentation in children in one primary school and one language school, after exposure to an adult with smear-positive pulmonary TB as well as the adherence to treatment.

## Population and Methods

### Index Case Investigation

The first index case (case 1) was a teacher in a preschool attended by 2.5–3.5-year-old children located in a municipality of Brussels. In December 2011, the diagnosis of smear-positive pulmonary TB was made after she had been coughing for 3 months. Culture testing for *Mycobacterium tuberculosis* and drug susceptibility testing (DST) revealed no drug resistance.

The second index case (case 2) was a teacher in a private language school, in another municipality of Brussels. Children aged 7–10 years attended his weekly language course, for 4–8 h a week. In April 2015, after having been ill for several months and coughed for at least 2 months, he was diagnosed with smear-positive pulmonary TB. Culture testing for *M. tuberculosis* and DST revealed isoniazid resistance.

### Contact Investigation

A contact investigation was carried out in the index households, extended families and in the two schools. Risk environment for transmission is defined as enclosed indoor spaces: a living space where people eat together, a bedroom, and also a classroom. Household contact and family were considered as close contacts; contacts that shared the same classroom, friends, and colleagues were considered as casual contacts, and other contacts attending the same school were considered as community contacts (9, 10). Each individual who had contact with an index case has been evaluated at least 8 weeks after the last notified exposure. This length of time, named “window period,” also called pre-allergic period, is the interval between acquisition of infection with *M. tuberculosis* and the point in time when an immunologic response becomes measurable: ≥8 weeks after the last relevant exposure to the index case (8, 9). The screening tool used in these investigations was the TST by intradermal injection (Mantoux technique) of 2 international units of tuberculin RT-23 [commercial Tuberculin Purified Protein Derivative (PPD) produced from *M. tuberculosis*, standardized by the World Health Organization (WHO), Staten Serum Institute of Copenhagen, Denmark]. Reaction reading was done 72 h after testing. The reaction to the test was considered positive if the induration was ≥5 mm for children and ≥10 mm for adults in line with national guidelines (Table 1) (11, 12).

**Table 1:** General interpretation criteria of the tuberculin test^*^.

**Diameter of the induration**	**Interpretation**
<5 mm	**Negative**
5–9 mm	**Negative**
	**Positive** - in case of infection with HIV or severe immune deficiency - in young children < 5 years of age, who have recently been in contact with an infectious patient or who are immune-primed or who show a clinical manifestation of active tuberculosis
	**Doubtful** - in persons ≥ 5 years of age: in case of close contact with an infectious tuberculosis patient - in children < 5 years of age or in persons ≥ 65 years of age: in the absence of risk factors
10–17 mm	**Positive** - in case of close contact with an infectious tuberculosis patient - and/or when there is an increased risk of infection or tuberculosis - in all children, regardless of their age
	**Doubtful** - in the absence of a risk factor - and/or in the case of BCG
≥18 mm	**Positive**

* *Translation from “Recommendations regarding the prevention of tuberculosis in healthcare facilities” of the Belgian Superior Health Council. 2013 Report Number: HGR 8579 (11). BCG, Bacillus Calmette-Guérin; HIV, Human Immunodeficiency Virus*.

When TST was doubtful or positive after a Bacillus Calmette-Guérin (BCG) vaccination, an IGRA was proposed to increase specificity (13, 14). The European Center for Disease Prevention and Control (ECDC) guidance states that IGRA testing should not replace TST after exposure to an infectious TB case but may be used, in addition to TST, as part of an overall risk assessment. Any positive result was considered. TST or IGRA positive results for contacts at high risk of developing active TB, in particular children aged <5 years and older adults aged more than 65 years, were considered as infected and preventive treatment was proposed, without considering their BCG status. Contacts with positive screening who fell outside this high-risk group or who declined treatment were informed about the red flags symptoms of TB disease. For contacts with low risk, without close contact exposure, IGRA can be used to rule out false positive TST reactions caused by BCG vaccination with a two-step approach (15).

Contact investigation was performed by specialized staff (nurses and social workers) from the two centers of expertise of TB in Belgium: the French-speaking “Association for Respiratory Health and Tuberculosis Control” FARES (Fonds des Affections Respiratoires) and the Flemish-speaking association VRGT (Vlaamse Vereniging voor Respiratoire Gezondheidszorg en Tuberculosebestrijding). Both organizations worked in close collaboration with the national Hygiene Inspection Agency that organized several visits and information sessions in both schools. Considering the young age of the contacts in the preschool (case 1), it was decided to include all children, teachers, and non-teaching staff in a first contact tracing.

In the language school (case 2), contact screening was further extended because of the large number of infected children in the first round of screening.

For case 1, the time elapsed between TB diagnosis (T0) and screening was 1 week (T1) for household contacts and 10 days for school contacts. For case 2, the period for the first round of screening (T3) was 3 weeks for the school contacts and 7 weeks (T7) for the second round. If the initial TST was negative but done before the eighth week (T8) after the last contact with the index case, TST was repeated to look for conversion, after the end of this “window period.”

Children <5 years old with a negative TST result were advised to receive preventive treatment (chemoprophylaxis) with isoniazid until TB infection was ruled out by a second negative TST after the “window period.” Information leaflets were made available to pediatricians and family doctors. Contacts who presented with a positive TST result were asked to seek medical advice to rule out TB disease.

## Results

For both index cases, the contact cohort was divided in three groups according to the stone-in-the-pond principle: close contacts (index case household and extended family), casual contacts (index case class and neighbor class, colleagues, and friends), and community contacts (other individuals and colleagues at index case school) (10).

### Case 1

#### Close Contacts (Table 2)

A total of 22 family members were exposed, of whom four were between 0 and 5 years old. The index case's child, aged 2 years, was diagnosed with culture-confirmed pulmonary TB. The husband declined being skin tested; he had received a BCG vaccine as a child and his chest X-ray revealed normal. Another family member, a 5-month-old girl with no history of BCG, was diagnosed with primary infection, presenting with 19 mm TST induration and feverish with a normal clinical evaluation and chest X-ray. However, blood evaluation revealed an elevated sedimentation rate (19 mm/h) and she was diagnosed with an active TB disease. Two out of the 11 remaining adults had a positive TST, a normal chest radiograph and were diagnosed as LTBI. Advice for the treatment of children was followed for all children. No treatment was proposed to the adults, considering non-compliance and/or contraindications.

**Table 2:** Characteristics of contacts in case 1.

	**Close**	**Casual**	**Community**
	**contacts**	**contacts**	**contacts**
	***n* = 22**	***n* = 47**	***n* = 260**
Female sex	*n* = 11 (50%)	*n* = 18 (38%)	*n* = 136 (52%)
0–4 years old	*n* = 5 (23%)	*n* = 46 (98%)	*n* = 28 (11%)
5–15 years old	*n* = 5 (23%)	*n* = 0 (0%)	*n* = 210 (81%)
>15 years old	*n* = 12 (55%)	*n* = 1 (2%)	*n* = 22 (8%)
Relationship to index patient			
Offspring (2 years)	*n* = 1 (4%)		
Husband (>15 years)	*n* = 1 (4%)		
Other family member (>15 years)	*n* = 11 (50%)		
Other family member (5–15 years)	*n* = 6 (27%)		
Other family member (0–4 years)	*n* = 3 (14%)		
Pupil in index class (0–4 years)		*n* = 18 (38%)	*n* = 0 (0%)
Pupil in index school (0–15 years)		*n* = 28 (60%)	*n* = 238 (91%)
Colleague/friends (>15 years)		*n* = 1 (2%)	*n* = 22 (8%)
No TST performed, normal chest X-ray	*n* = 1 (4%)	*n* = 1 (2%)	*n* = 3 (1%)
Negative TST	*n* = 17 (78%)	*n* = 40 (85%)	*n* = 252 (97%)
Positive TST with BCG history	*n* = 0 (0%)	*n* = 0 (0%)	*n* = 2 (0.7%)
Latent Tuberculosis Infection	*n* = 2 (8%)	*n* = 4 (8%)	*n* = 2 (0.7%)
TB disease	*n* = 2 (8%)	*n* = 2 (4%)	*n* = 1 (0.4%)
Isoniazid prevention (negative TST and 0–4 years)	3/3 (100%)	17/40 (42%)	9/27 (32%)
Isoniazid treatment for latent TB	0/2 (0%)	4/4 (100%)	1/4 (25%)
Initiated treatment for TB disease	2/2 (100%)	2/2 (100%)	1/1 (100%)

*n, number of cases; BCG, Bacillus Calmette–Guérin; TB, Tuberculosis; TST, Tuberculin skin test*.

#### Casual Contacts (Table 2)

All 18 children (2.5–3.5 years old) from the case 1 class, 28 children (3.5–4.5 years old) from a neighboring classroom and one adult colleague were classified as casual contacts. Two out of the 18 children were diagnosed with pulmonary TB and two others with an LTBI. One child didn't undergo a TST but had a regular clinical evaluation and a normal chest X-ray. In the neighboring class, 2/28 children were diagnosed with an LTBI. In total, considering both classes and the colleague as casual contacts, 6/47 (13%) were infected, of whom 2/47 (4%) presented with TB disease. Children <5 years old with a negative TST result were advised to receive chemoprophylaxis with isoniazid as preventive treatment until TB infection was ruled out by a second negative TST after the “window period.” In the class of case 1 and her neighbor class, only 17/40 (42%) of the parent's children followed this advice.

#### Community Contacts (Table 2)

The other 238 children in the school, the 22 teachers and the non-teaching school staff were considered as community contacts. One 4-year-old boy was diagnosed with high probable pulmonary TB; cultures did not grow *M. tuberculosis*. His classroom was not directly linked to the index class, but all preschool children met together twice a day in a shared indoor playground.

A 10-year-old girl had a positive TST (28 mm) at the first screening test (T3). Her chest radiograph was normal. As this girl was previously BCG-vaccinated, IGRA was done to interpret the tuberculin test according to the Belgian guidelines (13). Both IGRA results at T3 and T8 were negative. She was considered as recently infected; treatment was offered but declined. She was carefully followed up to exclude the development of TB by her pediatrician up to date (2018). Also, in this population, children <5 years old with a negative TST result were advised to receive chemoprophylaxis with isoniazid as preventive treatment, until TB infection was ruled out by a second negative TST after the “window period.” Only 9/27 (32%) of the children's parents followed this advice. Among the adult community contacts, two adult colleagues of the index case, who shared the same break room daily for at least 1 h, became positive at T8, with normal chest X-ray results. Both were considered as recently infected; only one followed the advice to start treatment with isoniazid.

#### Pulmonary TB in Contacts

In summary, five children (two close contacts, two casual contacts, and one community contact) were diagnosed with pulmonary TB during the index investigation of case 1. All five presented minimal symptoms: fever and cough. Morning gastric aspirates were performed on 3 consecutive days, and all five children were smear-negative for *M. tuberculosis*. An isoniazid-rifampicin-pyrazinamide based treatment was initiated. One of the casual contacts had a positive molecular polymerase chain reaction (PCR) for *M. tuberculosis* on gastric aspirate. Two of them (a close contact and a casual contact) had a cultured-confirmed TB disease. *M. tuberculosis* isolates of case 1 and the two children were sent to the “Belgian Scientific Institute for Public Health (previously known as WIV-ISP).” Molecular characterization of bacteria from the *M. tuberculosis* complex is used for epidemiological purposes to analyze the spread of specific genotypes. Mycobacterial interspersed repetitive units (MIRU) were done to determine strain identification. Using the standard 24-locus MIRU method, the TB genotype confirmed that the teacher, her child and the pupil had TB disease of the same strain.

### Case 2

#### Close Contacts (Table 3)

The household of index case 2 consisted of his flat mate who was diagnosed with an LTBI. The teacher had no family in Belgium.

**Table 3:** Characteristics of contacts in case 2.

	**Close**	**Casual**	**Community**
	**contacts**	**contacts**	**contacts**
	***n* = 1**	***n* = 57**	***n* = 101**
Female sex	*n* = 0 (0%)	*n* = 26 (46%)	*n* = 56 (55%)
0–4 years old			*n* = 1 (1%)
5–15 years old		*n* = 50 (88%)	*n* = 93 (93%)
>15 years old	*n* = 1 (100%)	*n* = 7 (12%)	*n* = 7 (7%)
Relationship to index patient			
Household other (>15 years)	*n* = 1 (100%)		
Pupil in index class (5–15 years)		*n* = 50 (88%)	
Pupil in index school (5–15 years)			*n* = 92 (91%)
Colleague/friends (>15 years)		*n* = 7 (12%)	*n* = 9 (10%)
No screening performed		*n* = 2 (3%)	*n* = 4 (4%)
Negative TST		*n* = 32 (56%)	*n* = 94 (93%)
Latent Tuberculosis Infection	*n* = 1 (100%)	*n* = 17 (30%)	*n* = 3 (3%)
TB disease		*n* = 6 (11%)	
Initiated treatment for latent TB		14/17 (82%)	3/3 (100%)
Initiated treatment for TB disease		6/6 (100%)	

*n, number of cases; TB, Tuberculosis; TST, Tuberculin skin test*.

#### Casual Contacts (Table 3)

The teacher's casual contacts consisted of 50 children, aged 6–12 years (mean: 8 years), which he saw weekly during 4–8 h in little groups, and seven colleagues. As these children were older than 5 years of age, no chemoprophylaxis was proposed during the window period. Positive TST was observed in 23/57 (40%) casual contacts. The five adults were urged to see their family doctor; all of them had a chest X-ray that was considered normal. The 18 children with positive TST were referred to a pediatrician in a specialized pediatric unit. Six of them had an abnormal chest X-ray and were therefore considered as having a pulmonary TB. Morning gastric aspirates were performed on 3 consecutive days and they were all smear-negative and culture for *M. tuberculosis* was negative. These six children received a rifampicin-pyrazinamide-ethambutol treatment, as soon as the index case DST result (isoniazid resistance) became available. The 12 other children with positive TST were considered as LTBI and treated with isoniazid. When resistance to isoniazid was noticed at the index case 2, all contacts with a diagnosis of LTBI had to change their treatment to rifampicin, but only 14 of the 17 casual contacts followed these guidelines.

## Discussion

### An Estimation of Infectivity

Belgium is a country with a low TB burden; nevertheless, we describe in this article two cases of widespread TB transmission in a school, both in a similar setting (TB prevalence 44.7 and 46.3 cases per 100,000 inhabitants, respectively), with the same socio-economic profile.

Both index cases are teachers and many of the contacts are young children. Both outbreaks have been well-documented and had a reasonable number of children to compare the outbreaks.

Among the first index case of all close contacts under 5 years of age, 40% were infected. When looking at the infectivity rate of all close contacts together for case 1, a percentage of 16% TB infection (8% LTBI and 8% TB disease) was observed. At school contact investigation, 13% was infected after casual contact (4% of the children with a TB disease). In the community contacts, 1.1% were infected and 0.4% had TB disease.

The second index case only had one close contact, who was infected (100%), but among the casual contacts 40% of the children were infected, and 11% of the children presented with TB disease. Among the occasional contacts, 3% were infected. Although, we conducted a well-followed protocol of diagnostic techniques and a detailed follow-up of the contacts, the number of contacts is small to reach definitive conclusions, and numeric findings should be considered moderately.

One of the questions is why the language teacher (case 2) was much more contagious than the preschool teacher. Both teachers were ill with frequent cough for more than 2 months before diagnosis, had cavitary chest radiograph and abundant acid-fast bacilli on sputum smear. The room where case 2 worked was smaller than the classroom of the pre-school teacher (case 1). Case 2 was a teacher in the Arabic language; the origin of his pupils was from Arabic speaking countries. The children were also older and had more time to get in contact with TB in their community and acquire LTBI before. The municipal of case 1 is known with a high immigration population, but infected children in her class were all born in Belgium. Roberts et al. describe TB transmission in schools. Adult teacher exposure with a sputum-smear positive source case is reported to cause 28–52% of infected children in close contacts and 2.9–51% in more extensive contacts (16).

Using the classification of proximity appears to be arbitrary. We were not able to register the hours of contact, neither the difference in baseline characteristics (e.g., migration background, overseas travel, the level of education of parents). This gives room for speculations why infectivity was higher in case 2.

### TB Elimination and Secondary Cases

The management of childhood TB outbreaks is crucial in TB elimination (17). Outbreaks in schools are difficult as severe concerns, and even panic can be expected between parents, teachers, and media. Given the lack of public knowledge and lack of experience among primary health care providers, it is essential to inform everybody, without delay, especially about the risk factors in children. Prompt diagnosis can reduce the severity of illness in the patient and even prevent further school-based transmission (18, 19). In both settings described, new cases with pulmonary TB disease were diagnosed at an early stage. The exposed young children are not only vulnerable to develop a seriously life-threatening or disabling clinical presentation but can also become a source of infectivity, even with smear-negative sputum (20). However, young children have a paucibacillary disease. The pupil of case 1, a 2.5-year-old boy, had a culture proved pulmonary TB (identic strain as his teacher) with negative gastric aspirates smear results. His father had a positive TST, was previously vaccinated with BCG and had a positive IGRA test (18). This raises the question whether not all parents of the index class should have been tested. The index case was judged to be (particularly) infectious, given the contact with the parents each morning at the start of school.

### Low TB Burden Country but a Higher Incidence in a Big City

We are describing an outbreak in a low TB burden country. However, this should be put in the following perspective. The two municipalities in which these schools were located have a much higher incidence of TB than that reported for the whole country, i.e., 44.7 cases per 100 000 population in 2011 for index 1 and 46.3 cases per 100 000 population reported in 2013 for index 2. Both municipalities have a diverse socio-economic profile with very low taxable incomes. In Belgium, TB incidence remained almost constant between 2011 until now: 9–10 cases/100 000 population (21). A strong correlation has been observed between ranking on poverty indicators and TB incidences across the 19 districts in Brussels. The poorest communities showed incidences equal to those of some developing countries (22).

In Belgium, TB is a notifiable infectious disease. Contact screening, as well as index screening, are done to prevent further spread. Active programs with systematic entry screening for asylum seekers and all prisoners exist. However, people visiting friends and relatives (VFR) from their country of origin are also at risk for new TB infection and disease. They are invited for free of charge screening on a voluntarily base. Information folders with a screening invitation containing visual information, designed with the participation of the target group, are used. These leaflets explain how to recognize symptoms, where to go for screening and propose pre- and post-travel TSTs for free. Despite all efforts, infection control fails too often. We still need more tailored strategies. Improved access to public health care is essential, as more nurses, social workers, and health professionals in the community are crucial for an appropriate outpatient clinical follow-up.

TB occurs more frequently in big cities where people at risk are over-represented. Recommendations for TB control programmes in these cities exist and are known to work (23).

### Spectrum of TB Infection and Risk for TB Disease

Children with a positive TST result and no clinical arguments for the disease were considered as LTBI. The WHO's definition of LTBI is a state of a persistent immune response to stimulation by *M. tuberculosis* antigens without evidence of clinically manifested active TB. TST will only measure a persistent TB immune response but cannot differentiate latent infection with cured or treated infections; TST is also a poor predictor for progression to active TB. TB infection should be considered as a dynamic spectrum (24).

In a pediatric population, TB disease is more often a clinical diagnosis that cannot be confirmed by bacteriological cultures. The risk of progression to active disease is higher in infected individuals who belong to specific high-risk populations. Major risk factors for TB activation include recent contact with an infectious patient. For young people, especially children below 5 years old, the risk of evolution to an active TB disease is even more elevated, and actually, we only have these tests available to make decisions for treatment, after recent exposure (25). Our two outbreaks confirm the high risk for progression to active TB disease, as they only concern children who presented with a TB disease. The little cousin of case 1 was only 5 months old and had a positive TST with normal chest X-ray. She was feverish and had an elevated sedimentation rate. We define her as a TB disease; to our opinion, she was in a spectrum of TB closer to illness than an LTBI. She was treated accordingly.

### IGRA Testing

To refine the TST interpretation, especially in previously BCG-vaccinated patients, IGRA is proposed by the Belgian guidelines to provide additional information (13). IGRA is an *in vitro* blood test, detecting the release of interferon-gamma (IFN-γ) by circulating T cells following stimulation by antigens unique to *M. tuberculosis* (26). IGRA results are unaffected by previous BCG vaccination. In this setting, the commercially available QuantiFERON-TB Gold In-Tube (QFT) test was used, based on the *in vitro* release of IFN-γ in response to *M. tuberculosis*-specific antigens ESAT-6, CFP-10, and TB7.7. Other antigens are currently being developed as a diagnostic tool for LTBI. IFN-γ responses to the latency antigen Heparin-Binding Hemagglutinin (HBHA) can stratify LTBI subjects into different risk groups (27, 28).

Following ECDC guidance, only for low-risk children (without exposure), IGRA can be used to rule out false positive reactions caused by BCG vaccination or exposure to non-TB mycobacterium (NTM) by a two-step approach (15). The reliability of IGRAs among young children are not yet well-defined and increased frequency of indeterminate results in children <5 years limits its use in the detection of LTBI in these children (29). TST+/QFT– discordant results are common, and it remains uncertain if this constellation indicates TB infection or not (30). To overcome misdiagnosis in BCG-vaccinated young children, tested for LTBI, Pavic et al. recommended using both tests and considering the child infected if either or both are positive (31, 32). Farhat et al. observed 8.5% positive TST results attributable to BCG vaccination, a rate decreasing to 1% in children tested >10 years after vaccination (33).

The previously described 10-year-old girl with occasional contact with the teacher in case 1 presented with a very positive TST (28 mm) and was previously vaccinated with a BCG vaccine. IGRA was done in a two-step approach after a positive TST at inclusion and after the window period. Both IGRA results were negative (34). We considered her to be recently infected. Family was convinced that this child had a negligible contact and refused treatment. After a consultation, her pediatrician and her family decided not to treat her. Red flags symptoms of TB disease were explained to her and the family. This situation demonstrates Cruz observations of substantial variations in the interpretation of national and international guidelines that exist in LTBI management (35).

One of the reflections was that IGRA could have been used more systematically in these cohorts, although the possible inconvenience of a blood test for children and the need of two nurses should be considered, even if only one visit is required. Adapted blood samples should be obtained and sent to a specialized laboratory in time. This was not feasible in the setting of a large screening organized at school. On the other hand, the use of the TST is also confronted by difficulties, such as result interpretation, the need for a follow-up visit by the patient and false-positive results caused by cross-reaction with the BCG vaccine and with NTM (29).

### Treatment Options

We treated all infected children for TB disease prophylaxis, except the 10-year-old girl as previously discussed. The adults were not systematically treated, considering non-compliance or contraindications as judged by their family doctors, this is common practice in Belgium. The authors wonder whether it would be better to have this decision taken in reference centers for infectious diseases or to make the guidelines more unambiguous.

Treatment options recommended for LTBI include isoniazid for 6 or 9 months, a 3-month regimen of weekly rifampicin plus isoniazid, isoniazid plus rifampicin for 3–4 months, or rifampicin alone for 3–4 months. We opted for the 6-month treatment with isoniazid in our index 1 cohort. For the index 2 cohort, known as isoniazid-resistant; we prescribed a 4-month rifampicin treatment.

The WHO recommends that children, aged <5 years, after the first screening round with a negative TST result, should receive preventive treatment (chemoprophylaxis) with isoniazid until TB infection is ruled out by a second negative TST after the “window period.” The coverage of treated children was very disappointing (in the casual and occasional contact, respectively, only 32–42% of the parents procured this treatment). This, despite apparent information from school and FARES/VRGT, an instructive letter for the family doctor, an information evening organized by a nurse and pediatrician at the school for parents and an available mobile number of a pediatrician for additional information. Once TB infection seemed to be ruled out in the first screening round the prophylactic treatment seemed to be too abstract for parents, in this case, where there was no regular individual contact between parents and nurse or doctor.

## Conclusion

Children in contact with an adult smear-positive TB case are at high risk of transmission. In this paper, we describe outbreaks in two schools, after exposure to a teacher with pulmonary TB. Both index cases showed a transmission rate of, respectively, 13 and 40% in their classes at school, defined as casual contacts. We observed, as described in the stone-in-a-pond principle, that proximity of contact increased the risk of infection.

Early diagnosis is essential as young children are at high risk of developing TB disease. TB disease was observed in, respectively, 4 and 11% of all the casual contacts. Our two outbreaks confirm the elevated risk in children for progression to active TB disease, as only children presented with a TB disease. TB infected children as well as children with TB disease had good compliance to the recommended treatment.

The WHO recommends to screen all young children (<5 years old) who recently were in contact with a person affected by pulmonary TB and to initiate LTBI treatment even before infection can be demonstrated. This chemoprophylaxis should be continued until TB infection is ruled out by a second negative TST after a “window period” of 8 weeks.

Despite all this knowledge, only 32–42% of the children younger than 5 years were treated with the proposed chemoprophylactic treatment. This low compliance reflects the difficulty of communication to staff, parents, and children in a school outbreak. Multidisciplinary communication is extremely important in preventing panic and in motivating compliance of pediatric clinical advices. Notwithstanding our serious efforts in both school outbreaks, we failed to convince the parents and their family doctors about this necessary chemoprophylactic treatment. It is essential to develop a new approach for this vulnerable group of patients. Public health authorities play a role in raising public awareness about the risks of TB for young children. This approach should be applied and evaluated by a National TB Control Program (NTP). In Belgium, the federal and regional governments underestimate the effort needed to implement these recommendations. National TB Control Programmes are missing. The two regional TB-organizations (VRGT and FARES), together with expert physicians and patients, have been lobbying for such national plans for many years up to the highest levels of policymaking and Inter-ministerial Conferences on health (36).

## Ethical Statement

The study protocols (numbers P2011/113 and A2012/051) were approved by the Erasme-ULB and CHU Saint Pierre University Hospital Ethics Committees, Brussels, Belgium, and informed written consent was obtained from all parents. Considering it is the normal implementation of routine contact investigations, required by infectious disease control law, no ethical concerns have been raised.

## Author Contributions

SD, AD, and VD: conception or design of the work. SD and LG: organized the database. AD, MF, MW, and WA: contributed to collection of the data. SD and JL: analysis of data for the work. VT: analysis of radiology. JL, FMo, FMa, and WA: drafting the work or revising it critically for important intellectual content. SD: agree to be accountable for all aspects of the work in ensuring that questions related to the accuracy or integrity of any part of the work are appropriately investigated and resolved.

### Conflict of Interest

The authors declare that the research was conducted in the absence of any commercial or financial relationships that could be construed as a potential conflict of interest. The handling Editor declared a past collaboration with one of the authors JL.

## Abbreviations

BCG: Bacillus Calmette–Guérin
DST: Drug Susceptibility Testing
ECDC: The European Center for Disease Prevention and Control
FARES: Fonds des Affections Respiratoires
IGRA: Interferon gamma release assay
IFN-γ: Interferon-gamma
LTBI: Latent tuberculosis infection
MIRU: Mycobacterial interspersed repetitive units
NTM: non-tuberculous mycobacteria
QFT: QuantiFERON®-TB Gold In-Tube test
TB: tuberculosis
TST: Tuberculin skin test
VRGT: Vlaamse Vereniging voor Respiratoire Gezondheidszorg en Tuberculosebestrijding
WHO: World Health Organization.
